# An assessment of the European Patient Summary for clinical research: a case study in cardiology

**DOI:** 10.3389/fmed.2024.1481551

**Published:** 2024-11-07

**Authors:** Gokce Banu Laleci Erturkmen, Ali Anil Sinaci, Tuncay Namli, Machteld J. Boonstra, Karim Lekadir, Polyxeni Gkontra, Catherine Chronaki, Rhonda Facile, Rebecca Baker, Rebecca Kush

**Affiliations:** ^1^SRDC Software Research & Development and Consultancy Corporation, Ankara, Türkiye; ^2^Department of Cardiology, Amsterdam UMC Location University of Amsterdam, Amsterdam, Netherlands; ^3^Department of Medical Informatics, Amsterdam UMC Location University of Amsterdam, Amsterdam, Netherlands; ^4^Amsterdam Cardiovascular Sciences, Heart Failure & Arrhythmias, Amsterdam, Netherlands; ^5^Barcelona Artificial Intelligence in Medicine Lab (BCN-AIM), Facultat de Matemàtiques i Informàtica, Universitat de Barcelona, Barcelona, Spain; ^6^Institució Catalana de Recerca i Estudis Avançats (ICREA), Passeig Lluís Companys, Barcelona, Spain; ^7^HL7 Europe Foundation, Brussels, Belgium; ^8^Clinical Data Interchange Standards Consortium, Austin, TX, United States

**Keywords:** health ecosystem, secondary use of EHR data, clinical research, interoperability, common data model

## Abstract

**Introduction:**

The European Health Data Space (EHDS) initiative was launched to create a unified framework for health data exchange across Europe. Central to this initiative is the European Electronic Health Record Exchange Format, designed to achieve interoperability of electronic health record data across Europe. Despite these advancements, the readiness of current guidelines and implementations, such as the European Patient Summary, to support secondary use in clinical research, particularly in cardiology, remains underexplored.

**Methods:**

This study aims to evaluate the European Patient Summary guidelines and their implementations, specifically the HL7 FHIR International Patient Summary Implementation Guide, to determine their suitability for secondary use in clinical research. The focus is on identifying gaps and extensions needed to enhance the utility of the European Patient Summary for building artificial intelligence models in assisting heart failure management.

**Results:**

We selected two European Union-funded research projects, DataTools4Heart and AI4HF, that aim to reuse electronic health record data to develop artificial intelligence models for personalized decision support services for heart failure patients. We analyzed their clinical use cases and the specific data items required, and we compared these with the current European Patient Summary guidelines and provided a detailed gap analysis indicating similarities and required extensions. In our gap analysis, we also compared the needs of DataTools4Heart and AI4HF with the HL7 FHIR International Patient Summary Implementation Guide to assess the extensions needed to support clinical research.

**Discussion:**

The EHDS is a transformative initiative to establish a European health data ecosystem that supports healthcare delivery and clinical research. Our comparative analysis demonstrates that, with minor extensions, these guidelines have significant potential to facilitate access to electronic healthcare record data for the secondary use, particularly in training AI models. We advocate for the adoption of an International Patient Summary format as a semantically interoperable core set of data elements, which will enhance global clinical research efforts and improve patient outcomes through precision medicine.

## 1 Introduction

The COVID-19 pandemic has highlighted the critical importance of robust health data ecosystems and efficient data-sharing architectures. The demand for timely, accurate, and comprehensive health data storage and exchange became paramount as the world faced an unprecedented public health crisis. The pandemic exposed several weaknesses in existing health information systems, including fragmented data silos, a lack of interoperability, and inadequate data-sharing mechanisms. These challenges have highlighted the urgent need for interconnected health data systems that facilitate seamless data sharing across different platforms, regions, and sectors.

In response to these challenges, the European Council has recognized the urgency of enhancing health data ecosystems across Europe, leading to the emergence of the European Health Data Space (EHDS) initiative (1). This initiative is a pivotal step toward building a European Health Union. The EHDS aims to create a unified and secure environment for health data exchange, enabling seamless cross-border collaboration and improving healthcare delivery, research, and policymaking. The EHDS has two main purposes: (1) enabling the primary use of health data to support or provide direct individual healthcare delivery to ensure continuity of care for the patient and (2) facilitating the secondary use (or reuse) of health data. This secondary use can involve individual-level, personal and non-personal health data, and aggregated datasets—particularly those generated during healthcare provision—to support research, therapeutic and vaccine development, innovation, policy-making, and regulatory science.

Central to achieving the goals of the EHDS is the European Electronic Health Record Exchange Format (EEHRxF) (2). The EEHRxF, initially introduced in the European Commission recommendation of 2019, provides the technical specifications and guidelines necessary to achieve interoperability of electronic health record (EHR) data across Europe. The EEHRxF defines key datasets under key priority data categories, including patient summaries, electronic prescriptions/dispensations, laboratory measurements, medical imaging reports, and hospital discharge reports. The eHealth Network (eHN), established under Article 14 of Directive 2011/24/EU on the application of patients’ rights in cross-border healthcare, is co-chaired by Member States’ representatives, and the European Commission and provides guidance and recommendations to facilitate the cross-border exchange of health data within the European Union. The eHN has defined the European Patient Summary (EPS) guidelines (3) as an identifiable dataset of essential and understandable health information to ensure safe and secure healthcare. EPS is implemented in the eHealth Digital Service Infrastructure (eHDSI) using Health Level 7 (HL7) Clinical Document Architecture (CDA) within the scope of MyHealth@EU (4), one of the cornerstones of the EHDS to facilitate the cross-border exchange of health data within the European Union. An HL7 FHIR IPS IG for EPS is under development. The EPS will be aligned with ISO 27269: 2021 Health Informatics—International Patient Summary (5) to ensure compatibility whenever applicable. For the implementation of the upcoming guidelines (such as the Laboratory Report), eHN has chosen the HL7 Fast Healthcare Interoperability Resources (FHIR) standard (6). An HL7 FHIR-based implementation guide (IG) has also been provided for ISO 27269 International Patient Summary (IPS) specification (7). Systemized Nomenclature of Medicine (SNOMED) has made a free set of Systemized Nomenclature of Medicine-Clinical Terms (SNOMED CT) available as part of its Global Patient Set (GPS) to support the implementation of the IPS.

The provisionally approved Regulation on EHDS on 24 April 2024 by the European Parliament and the Council (8) will make the adoption of the EEHRxF mandatory for EHR systems operating in all Member States, and EHR systems will be CE marked. Consequently, funds from both the European Commission and Member States will be allocated to ensure the interoperability of EEHRxF-format data, including patient summaries, both within and between countries. This development presents a significant opportunity to enhance interoperability in health data exchange. Moreover, even if EEHRxF is explicitly mentioned only for the primary use of health data, it marks an important step toward enabling the secondary use of EHR data. The heterogeneity of data formats across health data silos has been a major barrier to the secondary use of EHR data, and the introduction of interoperable EHR systems is key to overcoming this challenge.

EHR data collected for primary care purposes are invaluable resources for clinical research (9–13). These records provide comprehensive, real-world insights into patient health, capturing a wide array of clinical variables. The rich datasets derived from EHR data enable researchers to design clinical studies while considering the standard of care when establishing eligibility criteria and facilitating patient recruitment, as well as conducting observational studies to identify patterns and uncover insights that can enhance patient care. Additionally, EHR data serve as a critical data source for training artificial intelligence (AI) models in predictive analytics, thereby improving the accuracy and efficacy of these models in forecasting health outcomes, personalizing treatment plans, and advancing precision medicine.

In the EHDS architecture, the secondary use of health data, including training data for AI model development, will be regulated and structured to protect privacy while fostering innovation. Through the EHDS, AI developers can access a catalog of available datasets, such as Electronic Health Records (EHRs), registries, biobanks, and other relevant health data repositories, by following a series of steps. First, the AI model developer must verify eligibility and register with an authorized institution, such as a Data Access Body or another governing authority within the EHDS. Afterward, the developer should submit a detailed application outlining the project’s purpose, including how the data will be used, ensuring it aligns with permissible secondary uses. Following an ethical and legal review to confirm compliance with EU regulations, the developer can access the metadata catalog once the secondary use application is approved. This catalog allows the browsing of metadata descriptions to identify suitable datasets for AI model training based on parameters such as population characteristics, health conditions, and clinical outcomes. Once the relevant dataset (s) are identified, the developer submits a formal access request, followed by the signing of a Data Sharing Agreement (DSA). Depending on the architecture (centralized or federated), access to de-identified data is granted either through a centralized platform or a federated system, where data remain with individual data holders but are accessible for processing. At this stage, agreeing on a common data model for secondary use becomes crucial. AI developers need a consistent data structure to efficiently process and prepare the data for model training, including tasks such as cleaning, normalizing, and extracting features. The adoption of a common data format, such as the European Patient Summary (EPS), would significantly facilitate this process. EHR systems already implementing EPS for primary use could easily share de-identified patient medical summaries as training data for AI models. This would allow AI developers to establish their data preparation and model validation pipelines with the assumption that a common data model is available in each EHDS Data Access Node. For EHRs that do not currently support EPS, data transformation pipelines (14, 15) can be employed to convert local formats into the EPS format.

While the adoption of a standardized format such as the EPS within the scope of the EHDS offers significant opportunities for clinical research, a comprehensive analysis of its practical value remains absent. In this study, we aim to assess the current EPS guidelines and their implementations to evaluate their readiness to meet the requirements of clinical research studies that specifically seek to reuse patient summaries. Given that the data requirements for clinical research are highly dependent on specific research questions, conducting a domain-independent study is challenging. We have decided to focus on the requirements of one of the vertical domains, clinical research studies in the cardiology domain, as an initial attempt to highlight the gaps. This analysis is intended to contribute to the ongoing European effort to establish the necessary infrastructure for enabling the secondary use of EHR data in the EHDS. By providing a gap analysis, we aim to identify how the existing IPS can be extended to maximize its utility for clinical research in cardiology.

## 2 Methods

For this assessment, we selected two ongoing EU-funded R&D projects—DataTools4Heart and AI4HF—that aim to reuse EHR data to develop AI algorithms for personalized decision support services for heart failure (HF) patients. These projects were selected because they collectively address a broad range of clinical research questions in cardiology, covering use cases across all stages of care delivery: primary, secondary, and tertiary care. We analyzed their clinical use cases and the specific data items required, and we compared these with the current EPS guidelines (3), which identify core data elements with some references to applicable standards. In our gap analysis, we also compared the needs of DataTools4Heart and AI4HF with the HL7 FHIR IPS Implementation Guide (7), which provides a directly implementable specification for patient summaries.

DataTools4Heart (DT4H) (16) is an R&D project funded by the European Union’s Horizon Europe Framework under Grant Agreement No. 101057849. DT4H develops innovative tools to enable EHR data interoperability, quality, and reusability in cardiology while ensuring privacy, thereby improving collaboration between clinical centers. The DT4H toolbox will be exploited by the clinical partners to reuse existing, currently difficult-to-access EHR data in clinical research studies. The overarching aim of the DT4H project is to assess treatment, referral pathways, and prognosis of HF patients across different European countries using a privacy-enhancing federated learning approach based on real-world data. To investigate the different complicating factors of HF treatment, three clinical sub-studies for patients with an HF encounter have been proposed:

1. To investigate associations between chronic kidney disease and hyperkalemia and medication prescribed on discharge from a hospitalization for acute HF.
2. To develop a prognostic risk score for patients with acute HF presenting at the emergency department.
3. To investigate referral pathways in patients with HF who are referred from another healthcare facility for HF complaints.

AI4HF (Trustworthy Artificial Intelligence for Personalised Risk Assessment in Chronic Heart Failure, Grant No. 101080430) (17), is an innovative initiative that harnesses the power of artificial intelligence (AI) to provide personalized risk assessment and care plans for individuals living with HF. It utilizes advanced AI algorithms, global collaboration, and a patient-centered approach with the ultimate aim of improving healthcare outcomes. In the project, integrative and trustworthy AI models for tailoring the management of HF patients are co-designed, developed, evaluated, and exploited. The three sub-studies mentioned above are also studied in AI4HF, along with two additional HF-focused studies: (1) identification of novel electrocardiogram (ECG) and cardiology magnetic resonance (CMR)-based features to characterize HF patient subgroups, and (2) predicting major adverse cardiac events/end-stage heart failure outcome in patients with non-ischemic dilated cardiomyopathy.

As both projects utilize real-world EHR data to develop AI models specifically for HF patients, we have established a common data model (CDM) to improve data interoperability while addressing data heterogeneity across European regions and cardiology units. The proposed CDM has been implemented by utilizing the HL7 FHIR standard in terms of data model and data access Application Programming Interfaces (APIs). Following the HL7 FHIR profiling approach, analyzing the requirements of each use case, a set of HL7 FHIR profiles, code systems, and value sets was developed and published (18). The effort was initiated in the DT4H project and continued in the scope of the AI4HF project. In this context, the CDM was examined and extended to address the needs of AI4HF, and it was later renamed the Common Data Model for Heart Failure Research.

We have analyzed the EPS guideline core data element list as well as HL7 FHIR IPS IG (further referred to as IPS IG) and compared this with DT4H/AI4HF CDM to assess whether patient summaries provided in these formats can be readily utilized by DT4H and AI4HF to seamlessly extend the training and validation data sets in the context of both projects.

## 3 Results

In the following sections, we have summarized the result of the gap analysis between DT4H/AI4HF CDM, EPS guidelines, and HL7 FHIR IPS IG. We have presented our assessment by grouping the similarities and differences of core data elements under the main EPS sections.

### 3.1 Patient information

The patient profile in the DT4H/AI4HF CDM is quite similar to both the IPS IG patient profile and the EPS core data element list. It is possible to map required data elements, namely identifier, birthdate, gender, and address, directly to the IPS IG Patient Profile.

In the DT4H/AI4HF CDM, the “death date” is defined. This data element is not included within the EPS core data element list; however, it is available in the IPS IG Patient Profile. In the DT4H/AI4HF clinical use cases, the “cause of death” of a patient is an important element. The DT4H/AI4HF CDM represents this via a specific HL7 FHIR observation profile, where the primary condition for death is represented with an ICD-10 code. However, we could not locate this data element in either the EPS Core data element list or IPS IG.

Finally, in the DT4H/AI4HF CDM, the “ethnicity” of the patient is also needed to calculate the patient’s cardiovascular (CVD) risk score and assess algorithmic fairness. Ethnicity is not explicitly included in the EPS core data element list or the IPS IG. The value set for this data element in the DT4H/AI4HF CDM is a limited set of SNOMED CT codes that have been selected to represent the following values: African, Asian, Caucasian, Hispanic, and Unknown.

### 3.2 Problem lists

The condition profile in the DT4H/AI4HF CDM maps to the IPS IG Problems and Past Illnesses sections. The core data elements identified in the EPS, i.e., “problem/diagnosis description,” “onset date,” and “end date” are also included in the DT4H/AI4HF CDM. The clinical status data element required in the DT4H/AI4HF CDM to express the status as active or resolved is represented as an optional element in the IPS IG. In the EPS core element list, medical problems are grouped as “resolved” and “ongoing” in different sections. In the DT4H/AI4HF CDM, we also have an optional “severity” element to express severity, which is also available in the IPS IG. This information is not included in the EPS guideline.

In the DT4H/AI4HF CDM, symptoms are represented with an observation profile, with a selected set of SNOMED CT codes as a value set to represent cardiology-related symptoms. In the EPS core data set or in the IPS IG, there is no specific data element reserved for symptoms; it is assumed that the symptoms are represented via a problem data element as well.

### 3.3 Medications

In the DT4H/AI4HF CDM, separate HL7 FHIR profiles have been created for medications administered within the hospital (medication administration) and medications taken by patients outside the hospital (medication statement). When compared to the EPS core data elements (including medication brand name, active ingredient, date of onset for treatment, dosage regimen, route of administration, and intended use), we see that most of the required attributes in the DT4H/AI4HF CDM are already covered. The only missing information in the EPS guideline is whether the medication relates to inpatient or outpatient medication administration. Finally, the “end date” of medications is not specified in the EPS. In the IPS IG, it is possible to utilize both medication administration and medication statement within the medications section, and both of them already cover these requirements.

### 3.4 Procedures

For representing procedures, the content of the DT4H/AI4HF CDM is slightly different than the EPS core data elements and IPS IG. The EPS and IPS IG Procedure profiles include limited data elements, such as procedure description/code, date, and body site, which are also included in the DT4H/AI4HF CDM. Along with these elements, CDM includes the “reason” to record indication, “status” to record whether the procedure is ongoing or completed, and “category” to record whether it is a diagnostic or surgical procedure. Additional optional elements are the “outcome” to record the success of the procedure and the “report,” reference to any report resulting from the procedure.

### 3.5 Vital signs

The core data elements available in the DT4H/AI4HF CDM in the Vital Signs Profile are equivalent to the IPS IG Vital Signs Profile, including vital sign code, value, date, and units. In the EPS, vital signs are represented under the Results category as Observations, which includes the required data elements listed above. In the DT4H/AI4HF CDM, vital sign tests are specified with specific LOINC codes, including body height and weight, BMI, body surface area, systolic and diastolic blood pressure, heart rate, and oxygen saturation. These are covered by the Vital Signs value set of HL7 FHIR, which is utilized in the IPS IG.

### 3.6 Results

There is a good match between the EPS Core data element set results, the IPS IG Observation Results: laboratory/pathology profile, and the DT4H/AI4HF CDM Lab Result profile data elements. In the DT4H/AI4HF CDM, in addition to the Lab Result profile, we have three specific profiles to record an electrocardiogram (ECG), echocardiogram (ECHO), and magnetic resonance imaging (MRI) results as observation profiles where specific ECG, ECHO, and MRI parameters are represented as components with a well-defined value set. These details are not available in the EPS core data element set or IPS IG. However, it is possible to represent these with the Observation Results: radiology (IPS) profile.

### 3.7 Social history

In the DT4H/AI4HF CDM, there is a specific profile for recording Smoking Status, which overlaps with the Tobacco Use Profile of the IPS IG. It also aligns with the Social History core data elements identified in the EPS.

### 3.8 Admission or discharge information or healthcare encounters

In the DT4H/AI4HF CDM, we require the list of patient encounters, and when possible, these data are referenced from the conditions, lab tests, and vital signs indicating the scope of these elements. It is also important to note admission and discharge dates. Encounter information is unavailable in the IPS IG and the EPS, although it is available in eHN Hospital Discharge Report (HDR) guidelines and ISO IPS.

In DT4H/AI4HF CDM within the Encounter Profile, we require basic data elements such as “start date,” “end date,” and “reason.” In addition, we also need to record the classification of patient encounters (e.g., patient encounter, emergency visit) via the class attribute of the base FHIR Encounter resource. Finally, in the DT4H/AI4HF clinical use cases, it is required to know where a patient was admitted from (physician referral, transfer) and, if discharged, the organization to which the patient is discharged. The admission source is represented via the admission/admit source attribute with a value set including codes such as “from accident/emergency department, physician referral, transferred from another hospital, general practitioner referral.” The location/organization to which the patient is discharged is represented via the admission/admit source attribute.

### 3.9 Allergies and intolerances

In the DT4H/AI4HF CDM, we have a specific profile for recording allergies and intolerances, which is very much aligned with the core data elements of the IPS IG and EPS. In the DT4H/AI4HF CDM, the “clinical status” attribute is required, while it is optional in the IPS IG.

### 3.10 Other elements required

In the DT4H/AI4HF clinical use cases, we need to know about the referral events in EHR to investigate referral pathways in patients with HF. Hence, in the DT4H/AI4HF CDM, we have a specific profile to record referral events, the HL7 FHIR Service Request Profile. The “Requester practitioner role,” the “Performer practitioner role,” and the “Reason” are important data elements in the DT4H/AI4HF CDM for the Referral Category.

In the DT4H/AI4HF clinical use cases, information about the patient’s employment status, income level, and socio-economic status is required. The DT4H/AI4HF CDM represent these via specific HL7 FHIR Observation profiles. Similarly, in the DT4H/AI4HF clinical use cases, it is required to know the New York Heart Association (NYHA) (19) class of the patient. The DT4H/AI4HF CDM represents this via a specific HL7 FHIR Observation profile. The EPS core element set and IPS IG are represented under the Functional Status Category.

Finally, since CDM focuses on the clinical research studies in the cardiology domain, in the DT4H/AI4HF CDM, we have also identified an extensible value set to represent the codes for conditions as a selected set of ICD-10 codes, codes for the medications as a selected set of ATC codes. In the DT4H/AI4HF CDM Lab Result Profile, we have identified several lab tests required for cardiology studies with the identified LOINC codes and units. These value sets are available online in DT4H/AI4HF CDM (18).

## 4 Discussion

As summarized in Table 1, within the patient information, problem list, and procedures categories, the DT4H and AI4HF projects require additional data elements not included in the EPS Core data element list. Most of these additional elements can be represented in the HL7 FHIR IPS IG. However, among the extended elements, only the “cause of death” and “ethnicity” elements are not profiled in the IPS IG.

**Table 1:** A summary of the DT4H/AI4HF extensions over EPS core data elements and availability of these extensions in HL7 FHIR IPS IG.

Data element category	DT4H/AI4HF extensions added over EPS core data element	Availability of these extensions in HL7 FHIR IPS IG
Patient information	The death date element is added (via the “deceased Date Time” element of the HL7 FHIR patient resource)	Included in the IPS patient profile
	A cause of death element is added (via a specific Observation Profile)	Not included
	Ethnicity is added (via an extension over the HL7 FHIR Patient resource)	Not included
Problem list	A severity element is added (via the HL7 FHIR Condition resource)	Included in the IPS Condition Profile
	Symptoms are represented via a specific Observation Profile (the problem data element in EPS)	Represented via IPS Condition Profile
	A specific value set is defined to identify critical symptoms for the cardiology domain	IPS Condition Profile defines a preferred value set (Value Set: Problems—IPS) as a subset of SNOMED CT codes
	A specific value set is defined to identify a critical diagnosis for the cardiology domain by selecting codes from ICD-10	
Medications	Medications administered within the hospital and medications taken by patients outside the hospital are represented separately (via Medication Administration Medication Statement profiles)	Possible to use both Medication Administration and Medication Statement within the Medications Section
	In EPS, it is proposed to use ISO IDMP identifiers and SPOR (Substances, Products, Organizations, Referential) reference implementation to code the data element of the medicinal product description In DT4H/AI4HF CDM, a specific value set is defined to identify critical medications for the cardiology domain by selecting codes from ATC	The IPS Medication Profile defines the preferred value set for coding medications by choosing a subset of SNOMED CT for the medicinal products. However, as an alternative, binding an ATC-based value set is also recommended
Procedures	A reason element is added (via the “reason” element of the HL7 FHIR Procedure resource)	It is not included in the IPS Procedure Profile, but it is possible to use the base HL7 FHIR Procedure profile within the Procedures section of IPS Composition, which includes these
	The status element is added (via the “status” element of HL7 FHIR Procedure resource)	
	A category element is added (via the HL7 FHIR Procedure resource)	
	An outcome element is added (via the HL7 FHIR Procedure resource)	
	A report element is added (via the report element of HL7 FHIR Procedure resource)	
	The procedure value set has been defined to identify critical procedures for cardiology domain DT4H/AI4HF use cases by selecting codes from ICD10-PCS	IPS Procedure Profile defines the preferred value set for coding procedures by choosing a subset of SNOMED CT
Vital signs	The vital signs value set has been established to identify critical vital sign tests relevant to DT4H/AI4HF use cases	Included
Results	A specific value set is defined to identify critical laboratory tests for the cardiology domain by selecting codes from LOINC	A specific extensible value set (Value Set: Results Laboratory/Pathology Observation) has been defined in IPA IG by selecting a large set of LOINC codes under the Laboratory class
	Specific Observation profiles to record ECG, ECHO, and MRI results are defined where specific ECG, ECHO, and MRI parameters are included by specifying codes from SNOMED CT and LOINC where possible	These can be practically represented via the Observation Results: radiology (IPS) profile. This profile defines an extensible value set for coded radiology measurement observations by selecting codes from SNOMED CT, LOINC, and DICOM. The value sets specified do not directly cover the DT4H/AI4H CDM value set. However, as this value set is practically extensible, it is still possible to represent these data elements within IPS
Social history	None	—
Encounters	An Encounter Profile has been added	Not included in HL7 FHIR IPS. However, it is included in ISO IPS and eHN HDR guidelines
Allergies and intolerances	None	—
Referral	A referral profile has been added	Not included

Two important missing data element categories are encounters and referrals. It is critical for DT4H/AI4HF research studies in the cardiology domain to collect information about admission and discharge data and referrals between healthcare services. Additionally, linking problems, lab tests, radiology results, and medications with corresponding encounters is essential for DT4H/AI4HF studies. Within encounter information, it is possible to express the admission source and discharge disposition, which, to a certain extent, can be utilized to understand referral pathways. Therefore, adding encounter information as a separate category within the EPS/IPS guidelines would significantly increase their value for clinical research. It should be noted that encounters are included in the eHN HDR guidelines and ISO IPS.

In the DT4H/AI4HF CDM, specialized value sets have been defined for problems (including diagnosis and symptoms), medications, lab tests, procedures, and vital signs. As depicted in Table 1, the defined preferred and extensible value sets in the IPS IG often cover these specialized value sets. However, these specialized value sets indicate a set of selected codes for ensuring interoperability and identifying the critical data that should be available for specific research studies.

We suggest that the extension of EPS with these elements, which have been identified as gaps in Table 1 and summarized in this section, would greatly increase the practical use of patient summaries as a potential source of data for clinical research studies. It should be noted that in this study, we have focused only on the particular needs of the cardiology domain, which is a limitation. Similar studies should be carried out in different vertical domains. EPS/IPS extensions can be coordinated as profiles focusing on the needs of specialized domains, such as cardiology, respiratory disease, and pediatrics. These domain-specific profiles are needed to ensure interoperability and data availability in patient summaries, enabling secondary use for clinical research.

It should be noted that studies have already been initiated to extend the European EHRxF to facilitate secondary use for clinical research. An important initiative in this respect is the xShare project (23), funded by the EU. It aims to expand the EHRxF to effectively share and use health data within the EHDS for continuity of care, public health, and clinical research. Studies have already been initiated to define an extended core data element set (IPS + R) that could streamline clinical research by directly leveraging standard healthcare data. Initial xShare activities have been focused on analyzing various IPS-related standards [i.e., ISO IPS (5), HL7 FHIR IG for IPS (7), EPS (3), IHE IPS (20) and USCDI (21)] and comparing them with the CDISC CDASH core data elements for research and key data elements identified through IMI EHR4CR (10) and EHR2EDC, as well as key public health data elements (PHIRI) (22).

The gap analysis presented in this study is shared with the xShare consortium. When we have collaboratively compared our gap analysis, we already see many overlaps: In their ongoing studies, the xShare project has also identified Encounter as an important missing data element category, which is also in line with our findings from the gap analysis. Similar to our findings, patient death date is also identified through xShare analyses as a potential addition to core data elements. In addition, an identifier for clinical research patients is proposed as a “research subject identifier” to maintain patient privacy for clinical research and observational studies. Another gap identified by xShare for EPS is the need to indicate whether the medication is ongoing or stopped. Finally, the xShare project has identified an important potential additional information category, Adverse Events, which was not directly required in DT4H/AI4HF clinical studies but would be critical in other clinical studies.

The findings of the DT4H, AI4HF, and xShare projects reinforce the benefits for patients of not only ensuring that adequate data is readily available from healthcare for research and public health but also that there be an effort to harmonize or align across the various IPS and EPS standards/documents. The next step for xShare is to assign terminology for the core data element set so that healthcare data can be semantically interoperable. The DT4H/AI4HF CDM work has provided valuable input in that context. xShare is a collaborative that intentionally includes six standards development organizations (SDOs). The greatest benefit to patients is for these SDOs to collaborate and agree on a single standard for patient summary data. This is an important step in the road to the adoption of an international patient summary format as a semantically interoperable core set of data elements to enhance global clinical research efforts and improve patient outcomes through precision medicine.

## 5 Conclusion

The EHDS represents a transformative initiative to establish a European health data ecosystem, fostering collaboration, enhancing healthcare delivery, and enabling secondary use of EHR data. The EHDS is also pivotal for advancing clinical research studies to develop AI models for personalized healthcare. By providing a robust and standardized framework for the secure and efficient sharing of EHR data across Europe, the EHDS enables researchers to access real-world rich, diverse, and comprehensive data sets for training and validating AI models. This will not only enhance the accuracy and reliability of AI-driven insights but also accelerate the development of personalized therapies, ultimately improving patient outcomes and advancing the field of precision medicine. In this paper, we have conducted a comparative analysis of the EPS and one of its implementations, namely the HL7 FHIR IPS IG, evaluating its potential to be used as a standard to access EHR data for training AI models in two existing research projects. We have concluded that with few extensions, the EPS as a part of the EEHRxF has great potential to facilitate accessing EHR data for secondary use purposes in cardiology research studies. In addition, we encourage the generation and adoption of an EPS that incorporates the work of the various Standards Development Organizations (SDOs) that focus on healthcare and research standards towards a single definitive core set of patient summary information for healthcare that can be leveraged for research and public health. Given that clinical research and its associated standards are global, the EHDS will most benefit patients if core summary health data is standardized and semantically interoperable across borders.

## Data Availability

Publicly available datasets were analyzed in this study. This data can be found here: https://github.com/DataTools4Heart/common-data-model.

## References

[1] European Commission. (2022). Proposal for a regulation of the European Parliament and of the Council on the European Health Data Space COM/2022/197 final. Available at: https://eur-lex.europa.eu/legal-content/EN/TXT/?uri=CELEX:52022PC0197. (Accessed July 29, 2024)

[2] European Commission. (2019). Recommendation on a European Electronic Health Record Exchange Format. Available at: https://digital-strategy.ec.europa.eu/en/library/recommendation-european-electronic-health-record-exchange-format. (Accessed July 29, 2024)

[3] eHealth Network. (2023). Guideline on the electronic exchange of health data under Cross-Border Directive 2011/24/EU Patient Summary. Available at: https://health.ec.europa.eu/document/download/e020f311-c35b-45ae-ba3d-03212b57fa65_en?filename=ehn_guidelines_patientsummary_en.pdf. (Accessed July 29, 2024)

[4] European Commission. Electronic cross-border health services. Available at: https://health.ec.europa.eu/ehealth-digital-health-and-care/electronic-cross-border-health-services_en. (Accessed July 29, 2024)

[5] ISO. (2021). ISO 27269:2021 Health informatics—International Patient Summary. Available at: https://www.iso.org/standard/79491.html. (Accessed July 29, 2024)

[6] MyHealth@EU Consortium. MyHealth@EU, Laboratory Report, Hl7 FHIR Implementation Guide. Available at: https://fhir.ehdsi.eu/laboratory/index.html. (Accessed August 12, 2024)

[7] HL7. International Patient Summary Implementation Guide 2.0.0-ballot—STU 2 Ballot. Available at: https://build.fhir.org/ig/HL7/fhir-ips/. (Accessed July 29, 2024)

[8] European Commission. European Parliament legislative resolution of 24 April 2024 on the proposal for a regulation of the European Parliament and of the Council on the European Health Data Space (COM(2022)0197—C9-0167/2022—2022/0140(COD)). Available at: https://www.europarl.europa.eu/doceo/document/TA-9-2024-0331_EN.html. (Accessed July 29, 2024)

[9] Laleci GB Yuksel M Dogac A. Providing semantic interoperability between clinical care and clinical research domains. IEEE J Biomed Health Inform. (2013) 17:356–69. doi: 10.1109/TITB.2012.2219552 23008263

[10] De Moor G Sundgren M Kalra D Schmidt A Dugas M Claerhout B. Using electronic health records for clinical research: the case of the EHR4CR project. J Biomed Inform. (2015) 53:162–73. doi: 10.1016/j.jbi.2014.10.006, PMID: 25463966

[11] Sinaci AA Laleci Erturkmen GB. A federated semantic metadata registry framework for enabling interoperability across clinical research and care domains. J Biomed Inform. (2013) 46:784–94. doi: 10.1016/j.jbi.2013.05.009, PMID: 23751263

[12] Yuksel M Gonul S Laleci Erturkmen GB Sinaci AA Invernizzi P Facchinetti S. An interoperability platform enabling reuse of electronic health records for signal verification studies. Biomed Res Int. (2016) 2016:6741418. doi: 10.1155/2016/6741418, PMID: 27123451 PMC4830705

[13] Asselbergs FW Kotecha D. The CODE-EHR global framework: lifting the veil on health record data. Eur Heart J. (2023) 44:3398–400. doi: 10.1093/eurheartj/ehad424, PMID: 37408471

[14] Namli T Anıl Sınacı A Gönül S Herguido CR Garcia-Canadilla P Muñoz AM. A scalable and transparent data pipeline for AI-enabled health data ecosystems. Front Med. (2024) 11:1393123. doi: 10.3389/fmed.2024.1393123, PMID: 39139784 PMC11321077

[15] Sinaci AA Gencturk M Teoman HA Laleci Erturkmen GB Alvarez-Romero C Martinez-Garcia A. A data transformation methodology to create findable, accessible, interoperable, and reusable health data: software design, development, and evaluation study. J Med Internet Res. (2023) 25:e42822. doi: 10.2196/42822, PMID: 36884270 PMC10034606

[16] Data Tools4Heart Project Consortium. DataTools4HeartPreoject, A European Health Data Toolbox for Enhancing Cardiology Data Interoperability, Reusability and Privacy. Available at: https://www.datatools4heart.eu/. (Accessed July 29, 2024)

[17] AI4HF Consortium. AI4HF Project, Trustworthy Artificial Intelligence for Personalised Risk Assessment in Chronic Heart Failure. Available at: https://www.ai4hf.com/. (Accessed July 29, 2024)

[18] DT4H Consortium. DataTools4Heart Common Data Model Specifications. Available at: https://github.com/DataTools4Heart/common-data-model). (Accessed July 29, 2024)

[19] White PD Myers MM. The classification of cardiac diagnosis, with especial reference to etiology. Am Heart J. (1925) 1:87–95. doi: 10.1016/S0002-8703(25)90008-7

[20] Integrating Healthcare Enterprise. IHE International Patient Summary (IPS) Profile. Available at: https://wiki.ihe.net/index.php/International_Patient_Summary_(IPS). (Accessed August 12, 2024)

[21] Office of the National Coordinator for Health IT. United States Core Data for Interoperability (USCDI). Available at: https://www.healthit.gov/isp/united-states-core-data-interoperability-uscdi. (Accessed August 12, 2024)

[22] PHIRI Consortium. The Population Health Information Research Infrastructure. Available at: https://www.phiri.eu/. (Accessed August 12, 2024)

[23] xSHare Consortium. xShare Project, Expanding the European EHRxF to share and effectively use health data within the EHDS. Available at: https://xshare-project.eu/. (Accessed August 12, 2024)

